# Tuning the Locally Enhanced Electric Field Treatment (LEEFT) between Electrophysical and Electrochemical Mechanisms for Bacteria Inactivation

**DOI:** 10.1021/acs.est.4c00503

**Published:** 2024-08-06

**Authors:** Ting Wang, Xing Xie

**Affiliations:** † School of Civil and Environmental Engineering, Georgia Institute of Technology, Atlanta, Georgia 30332, United States; ‡ Institute for Electronics and Nanotechnology, Georgia Institute of Technology, Atlanta, Georgia 30332, United States

**Keywords:** locally enhanced electric field treatment, electroporation, electrochemical oxidation, bacteria inactivation, water disinfection

## Abstract

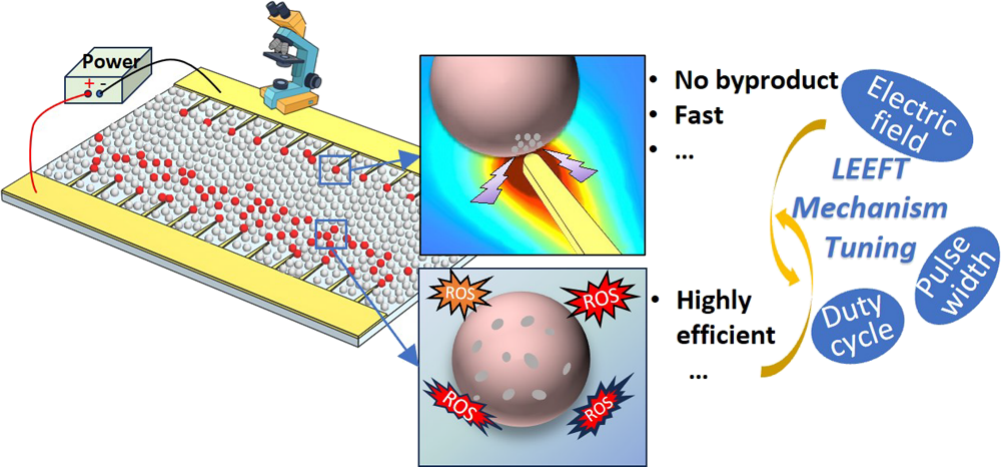

Efficient drinking water disinfection methods are critical for public health. Locally enhanced electric field treatment (LEEFT) is an antimicrobial method that uses sharp structures, like metallic nanowires, to enhance the electric field at tips and cause bacteria inactivation. Electroporation is the originally designed mechanism of LEEFT. Although oxidation is typically undesired due to byproduct generation and electrode corrosion, it can enhance the overall disinfection efficiency. In this work, we conduct an operando investigation of LEEFT, in which we change the electrical parameters to tune the mechanisms between electrophysical electroporation and electrochemical oxidation. Pure electroporation (i.e., without detectable oxidation) could be achieved under a duty cycle of ≤0.1% and a pulse width of ≤2 μs. Applying 2 μs pulses at 7–8 kV/cm and 0.1% duty cycle results in 80–100% bacteria inactivation with pure electroporation. A higher chance of oxidation is found with a higher duty cycle and a longer pulse width, where the antimicrobial efficiency could also be enhanced. For water with a higher conductivity, a higher antimicrobial efficiency can be achieved under the same treatment conditions, and electrochemical reactions could be induced more easily. The findings shown in this work improve the fundamental understanding of LEEFT and help optimize the performance of LEEFT in real applications.

## Abstract 2

The antimicrobial mechanism of locally enhanced electric field treatment (LEEFT) is tuned between electroporation and electrochemical oxidation, making it applicable for different types of water disinfection.

## 1 Introduction

Unsafe water sources are responsible for 1.2 million deaths globally each year. In 2020, there was still 26% of the world population who had no access to safe drinking water.^1^ Therefore, seeking an efficient water disinfection method is of great significance. Locally enhanced electric field treatment (LEEFT) is a novel antimicrobial method, where electrodes decorated with sharp structures, such as metallic nanowires, are used to generate a locally enhanced electric field for bacteria inactivation. When cells are exposed to a strong electric field, electroporation is induced to form pores on the lipid bilayer membrane, which can lead to cell inactivation. Electroporation is an electrophysical process resulting from the movement of charged ions, and it does not involve charge transfer (i.e., redox reactions) that generates chemical byproducts. Studies have demonstrated that electroporation is induced in LEEFT on both positive and negative electrodes and can cause ultrafast bacteria inactivation with nanosecond electrical pulses.^2,3^ In addition to electroporation, electrochemical reactions may also be induced by electrical pulses. These reactions are typically undesired in LEEFT since they may generate byproducts and/or cause electrode corrosion, which lead to secondary contaminants in treated water.^4^ Nevertheless, the reactive species generated from these electrochemical reactions, primarily oxidation reactions, may kill bacteria and improve the overall disinfection efficiency. Therefore, based on requirements of different applications, it is desired to rationally tune the mechanism of LEEFT between electrophysical (electroporation) and electrochemical (oxidation) processes. If we can precisely control these mechanisms, we may intentionally introduce some oxidation in LEEFT to improve the disinfection efficiency while maintaining minimum and acceptable byproduct generation.

The combination of electroporation and electrochemical oxidation has been studied for applications such as water disinfection. Electrochemically generated active chlorine and oxidative species were combined with electroporation for water disinfection using GO-MMO (graphene oxide-modified mixed metal oxidation) or a SnO_2_-coated anode. The antimicrobial efficiency under 2 V DC (direct current) was increased from <2 log by only electroporation to 6 log by the combined mechanism.^5,6^ Chen and group reported electric field water disinfection using Cu-based electrodes, where ROS accounted for ∼20% of bacteria inactivation when the total inactivation was around 90%.^7−9^ Electroporation and electrochemical oxidation were also coupled for eliminating antibiotic-resistant genes (ARGs). The combination of electroporation and oxidation increased bacteria damage and inactivation, which promoted the reactive species diffusion into the cells and ARG leakage from the cells, so that reactive chlorine and oxygen species can degrade ARGs more effectively.^10^ The synergistic effect of electroporation and simple chemical oxidation is also investigated, such as combining electroporation with added ozone, where electroporation can damage the cell membrane and facilitate ozone diffusion into cells.^11^

When a potential difference is applied between positive and negative electrodes and the voltage across the electrical double layer at the electrode–electrolyte interface exceeds a threshold value, electrochemical reactions could be induced. After a pulse starts, it takes some time for the threshold to be reached since the double layer is charged as a capacitor. The time for the double layer being charged to the threshold voltage is called the threshold time *t*_th_*(s)* and can be calculated as
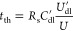
where *R*_s_ (Ω) is the solution resistance between the two electrodes, *C*_dl_^′^ (F) is the capacitance of the series connection of the two double layer capacitors, *U*_dl_^′^ (V) is the threshold voltage across both double layer capacitors in series, and *U* (V) is the applied voltage across the two electrodes.^4^ The threshold voltage, over which electrochemical oxidation reactions can be triggered, depends on several factors, including the electrode material, temperature, pH, chemical content of the fluid, and types of electrochemical reactions.^12,13^ Therefore, the electrochemical reactions can be affected by many factors, such as the pulse width, electrode material, electrolyte properties, and applied voltage.^14^ In addition, the charges that have been built up in the electrical double layer may not be fully discharged between pulses and could accumulate; therefore, the pulse direction and frequency will also affect the electrochemical reactions. The general methods to minimize undesired electrochemical reactions include using electrode materials having a larger double layer capacity, applying bipolar, nonsymmetrical, low-frequency, and short pulses, and lowering the medium conductivity if possible.^4,15−17^

In this work, the mechanism of LEEFT is tuned between the electrophysical process (electroporation) and the electrochemical process (oxidation) by adjusting the electrical parameters, including the electric field, pulse width, frequency, and duty cycle (i.e., the ratio of the pulse width to the period). The antimicrobial efficiency and oxidative stress are evaluated using a model bacteria *Staphylococcus epidermidis* (*S. epidermidis*) on a lab-on-a-chip device. The effect of the medium conductivity on the antimicrobial mechanism tuning is studied. The different patterns of bacteria inactivation induced by electroporation and oxidation are also investigated and compared. The results and trend obtained from the study with an inert electrode material (gold) and a simple water matrix can serve as a baseline to guide the mechanism tuning in other conditions and applications.

## 2 Materials and Methods

### 2.1 Lab-on-a-Chip Fabrication and Electric Field Simulation

To visualize and characterize the antimicrobial mechanism in LEEFT, a lab-on-a-chip was fabricated using an e-beam lithography and lift-off method based on previous studies (Figure S1).^2,3^ Briefly, gold electrodes with gold nanowedges on the edge were deposited on a glass wafer to make the chips. The gap between the positive and negative electrodes was 25, 50, or 100 μm. Gold nanowedges are 8 μm long and 200 nm wide at the tip. There are 330 nanowedges on one chip for antimicrobial efficiency characterization. The fabricated chips were coated with poly-l-lysine (Sigma-Aldrich) for bacteria immobilization. To reuse the chips, the used chips were washed with 5% bleach, 30% H_2_O_2_, and deionized (DI) water sequentially and recoated with poly-l-lysine.

### 2.2 Bacteria Culture, Harvest, and Immobilization on the Chip

*S. epidermidis* (ATCC 12228) was used as a model bacterial strain in this study. It is a commonly used model bacteria strain in microbiology studies, and its round and regular shape allows easier image processing of the data acquisition. The bacteria culture and harvest methods are discussed in our previous paper.^2^ Briefly, *S. epidermidis* was cultured in nutrient broth at 35 °C, then harvested and concentrated by centrifuging in 10 mM phosphate buffer for 3 times. To immobilize the cells on the chip, a drop of the prepared bacteria solution was added onto a poly-l-lysine-coated chip to cover the electrode gap and allowed immobilization for 50 min. Then, the unattached cells were gently washed away with 5 mL of DI water using a pipet. After adding a drop of the medium containing the live/dead cell-distinguishing stain, the chip was flipped, secured on a coverslip, and loaded onto an inverted microscope for observation. DI water or 6.7 mM Na_2_SO_4_ were used as the medium in this work, which have conductivities of 0.5 and 1500 μS/cm, respectively.

### 2.3 Electrical Pulses and the Antimicrobial Efficiency

The electrical pulses were applied to the chip by using a pulse generator (Avtech Electrosystems, AV-1010-B), which was triggered by a waveform generator (Keysight, 33509B). The pulse waveform and voltage across the electrodes were measured using an oscilloscope (Tektronix, DPO 5104). The applied electric field used in the figures and discussions is the background electric field, which is calculated by the equation EF = *V*/*d*, where *V* is the applied voltage and *d* is the distance between the positive/negative electrodes. Since the electrode gap is subject to change in other studies and real applications, using a background electric field to represent the applied treatment strength makes it more convenient to compare between different studies. The electric field enhancement by the nanowedge and the distribution on the chip were simulated using COMSOL Multiphysics. The detailed method is described in our previous paper.^3^ The relationship between the applied (background) electric field and the enhanced electric field at the nanowedge tip is shown in Figure S2. The enhanced electric field at 0.1 μm from the nanowedge tip is enhanced about 7 times compared to the applied electric field.

The antimicrobial efficiency of LEEFT is represented by the percentage of nanowedges inducing bacteria damage or inactivation around the tips. Since LEEFT is a heterogeneous process, the area of interest (effective zone) is the nanowedge tips. Therefore, the percentage of the nanowedges that can induce bacteria damage/inactivation is the best characterization of the antimicrobial efficiency of LEEFT.^3^ When referring to the total number of inactivated bacteria on the electrode surface instead of specifically at tips, we use other phrases, such as overall disinfection efficiency, to distinguish.

Three parameters were tuned in the experiments: the applied electric field, the pulse width, and the duty cycle (the ratio of the pulse width to the period). The parameters are explained graphically in Figure S3. The performance of LEEFT was tested under three pulse widths, 20 ns, 2 μs, and 200 μs and three duty cycles, 10, 0.1, and 0.001%, making the period range from 200 ns to 20 s and the frequency range from 5 MHz to 0.05 Hz. According to our previous study, bacteria inactivation by electroporation is very fast in LEEFT, and the inactivation efficiency could reach a plateau after an effective treatment time (pulse width × pulse number) of 20 ms.^2^ Therefore, the effective treatment time of 20 ms is kept for all treatment conditions, which is controlled by applying a fewer number of pulses for a longer pulse width (i.e., 10^6^, 10^4^, and 10^2^ pulses for a pulse width of 20 ns, 2 μs, and 200 μs, respectively). The total treatment time is defined as the product of the period and the pulse number. The DC (direct current) treatment lasted for 2 s, which can be considered a single 2 s pulse with 100% duty cycle, 2 s effective treatment time, and 2 s total treatment time.

### 2.4 Double Staining Method, Oxidative Stress Detection, and Microscopy

Due to the possibility of reversible electroporation and bacteria revival, we used a double staining method to determine reversible bacteria damage and permanent inactivation. The damaged bacteria were first stained using 5 μM SYTOX Green (Invitrogen). After 20 min, 15 μM propidium iodide (PI) (Invitrogen) was used to stain the inactivated cells. The details are explained in the Supporting Information (Figure S4).^18^ Other potential reasons causing cell fluorescence, such as electrophoresis of the stain or heat damage, have been ruled out due to the same phenomenon on both positive and negative electrodes and the negligible temperature change from our simulation results (data not shown).

The oxidative stress in bacteria cells was measured by a cell membrane-permeable fluorescence probe DCFH-DA (Sigma-Aldrich).^2,3^ Cells were first stained with 0.2 mM DCFH-DA for 50 min during cell immobilization. When electrical pulses were applied, the cells having oxidative stress showed green fluorescence. Both the fluorescence intensity and the area of cells showing fluorescence are positively correlated with the oxidation strength. Therefore, the overall cell oxidative stress was quantified by the product of the area of cells showing fluorescence and the mean DCFH-DA fluorescence intensity of the cells. To demonstrate if cell inactivation was due to oxidation, an oxidation scavenger dimethyl sulfoxide (DMSO) was added at 20% before electrical pulses were applied to quench the oxidation effects. Due to the relatively low concentration and short contact time, DMSO does not cause cell damage and thus will not interfere with the experimental results.

The bacteria were observed using an inverted fluorescence microscope (Zeiss Axio Observer 7). The cell and nanowedge images were captured via the differential interference contrast (DIC) channel. PI was excited at 555 nm. SYTOX Green and DCFH-DA were excited at 488 nm. All emission light was filtered with a Zeiss 90 HS filter.

### 2.5 Data Analysis

All microscopy images were processed by using scripts developed in MATLAB (version 2021b, MathWorks). Each treatment condition was repeated three times with three chips. The error bars represent the standard deviations from the three independent replicates.

## 3 Results and Discussion

### 3.1 Indication of Bacteria Inactivation and Oxidative Stress

**Figure 1:**
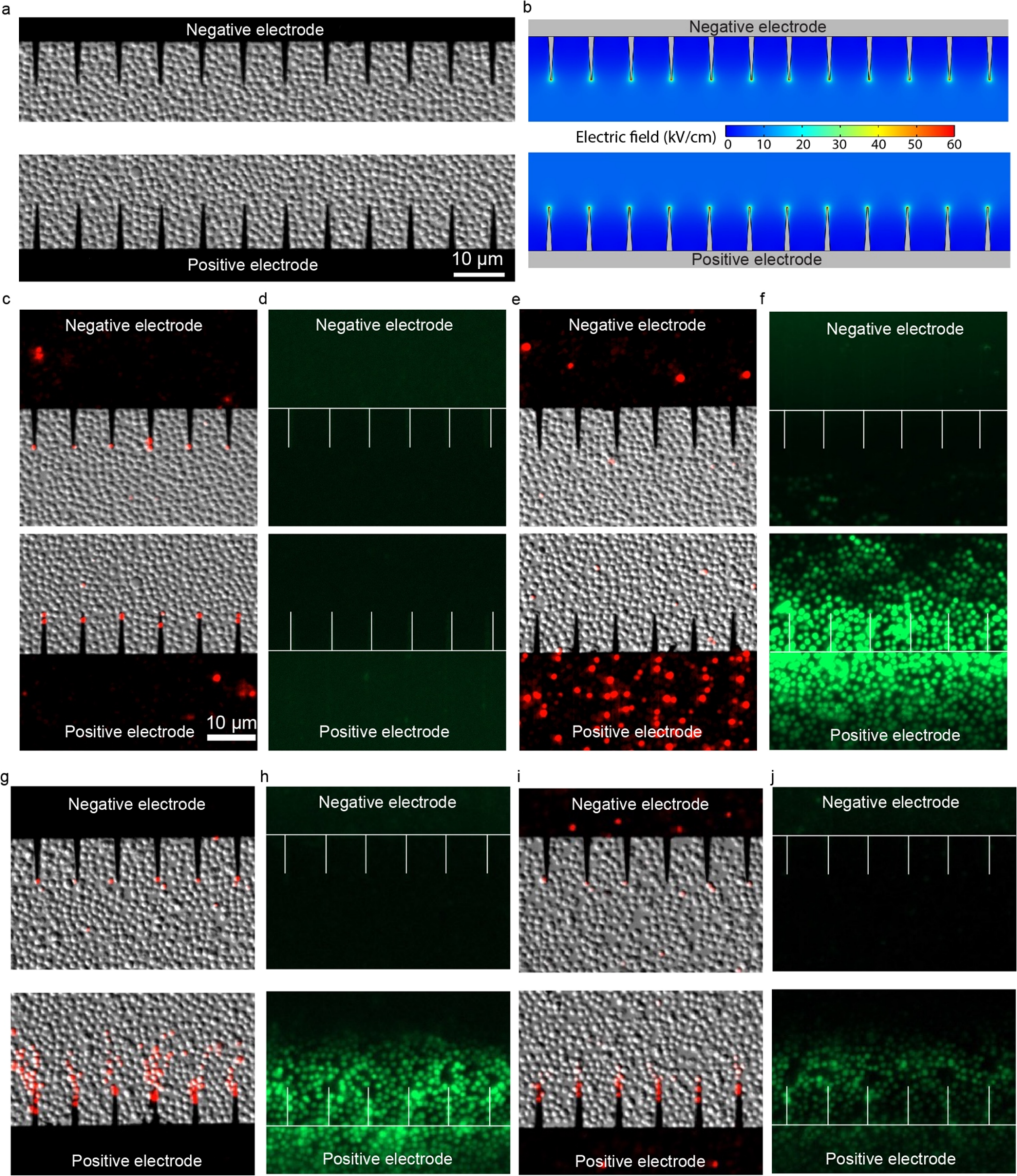
Electroporation and electrochemical oxidation on the chip. (a) *S. epidermidis* immobilized on the chip surface before treatment. (b) Simulation of the locally enhanced electric field when the applied electric field is 6 kV/cm. (c, d) Ten thousand 2 μs pulses are delivered at 8 kV/cm and 0.1% duty cycle to achieve electroporation. (c) Bacteria at nanowedge tips are inactivated. (d) No oxidative stress is detected by DCFH-DA. (e, f) Single 2 s pulse at 1 kV/cm is applied to achieve oxidation. (e) Cells are randomly inactivated on the surface of the positive electrode. (f) Significant oxidative stress is detected in cells around the positive electrode area. (g, h) A hundred 200 μs pulses are delivered at 8 kV/cm and 0.1% duty cycle to achieve the combination of electroporation and oxidation. (g) Group of cells are inactivated above nanowedge tips at the positive electrode. (h) Significant oxidative stress is detected. DMSO is added at 20% to quench oxidation at the same treatment as in panels (g) and (h). (i) Bacteria above nanowedges are protected by DMSO against oxidation. (j) Oxidative stress is largely quenched by DMSO.

To better distinguish bacteria inactivation by electroporation (the electrophysical mechanism) and oxidation (the electrochemical mechanism) in LEEFT, operando investigation has been conducted to observe the bacteria inactivation pattern and measure the oxidation level. Figure 1a shows a monolayer of model bacteria *S. epidermidis* immobilized on the chip surface before treatment. When a voltage is applied to the two electrodes, a nonuniform electric field will be formed with a much higher strength at the nanowedge tips, on both positive and negative electrodes, as shown in Figure 1b.^2^ This local electric field enhancement is due to the lightning rod effect. Bacteria inactivation is indicated by the red fluorescence of PI staining since the PI stain can only enter dead cells with the compromised cell membrane and then bind to DNA and show an enhanced fluorescence. The oxidation level inside cells is detected by the DCFH-DA stain, which shows a green fluorescence under oxidative stress.^19^ When electroporation is the dominant mechanism, the bacteria very close to the nanowedge tips are inactivated on both electrodes (by showing a red fluorescence of PI, as shown in Figure 1c), and no oxidative stress is detected by DCFH-DA (no cell shows a green fluorescence, as shown in Figure 1d). When electrochemical oxidation is the only mechanism, bacteria on the positive electrode surface are randomly inactivated (Figure 1e), which is different from electroporation at the nanowedge tip. The randomness is caused by the different resistances of the bacteria to oxidative stress. Although the tips may intensify electrochemical reactions at some level, due to the small surface area, oxygen species generated from the tips may diffuse away or be diluted by surrounding water very quickly, therefore not leading to significant cell inactivation at the tips. Significant oxidative stress could be detected by DCFH-DA around the whole positive electrode area (Figure 1f). Under DC, the gold electrode is not likely to induce many reactive oxygen species (ROS) due to a high likelihood of oxygen evolution. So, the oxidative stress generated here could be due to dissolved oxygen or direct oxidation.

When electroporation and electrochemical oxidation both take place, more bacteria are inactivated randomly above the nanowedges at the positive electrode (Figure 1g), and oxidative stress could be detected (Figure 1h). When the applied electric field is 8 kV/cm, the voltage applied to the electrodes is 40 V. Although ROS cannot be easily generated under DC, as discussed above, it is possible that with short pulses at a high applied voltage (e.g., 200 μs at 40 V), intermediate ROS are generated before oxygen evolution, thus causing oxidative stress without seeing oxygen bubbles. The divergent shape of the dead cells could be due to the diffusion of oxidative species from the nanowedge tip. When 20% oxidation scavenger DMSO is added under these conditions, the oxidative species could be largely quenched (a weaker fluorescence, as shown in Figure 1j), and the bacteria could be protected from oxidation (fewer dead cells above nanowedges), while the bacteria at the nanowedge tips killed by electroporation are not affected (Figure 1i). Compared to Figure 1g, more bacteria on the electrode surface are inactivated in Figure 1e. Since electroporation only kills bacteria at nanowedge tips, the bacteria inactivation on the positive electrode surface is only due to oxidation. This phenomenon indicates that oxidation induced by a single 2 s pulse at 1 kV/cm (Figure 1e) is more severe than that induced by a hundred 200 μs pulses at 8 kV/cm (Figure 1g).

### 3.2 Electroporation, Electrochemical Oxidation, and the Combination of Both

**Figure 2:**
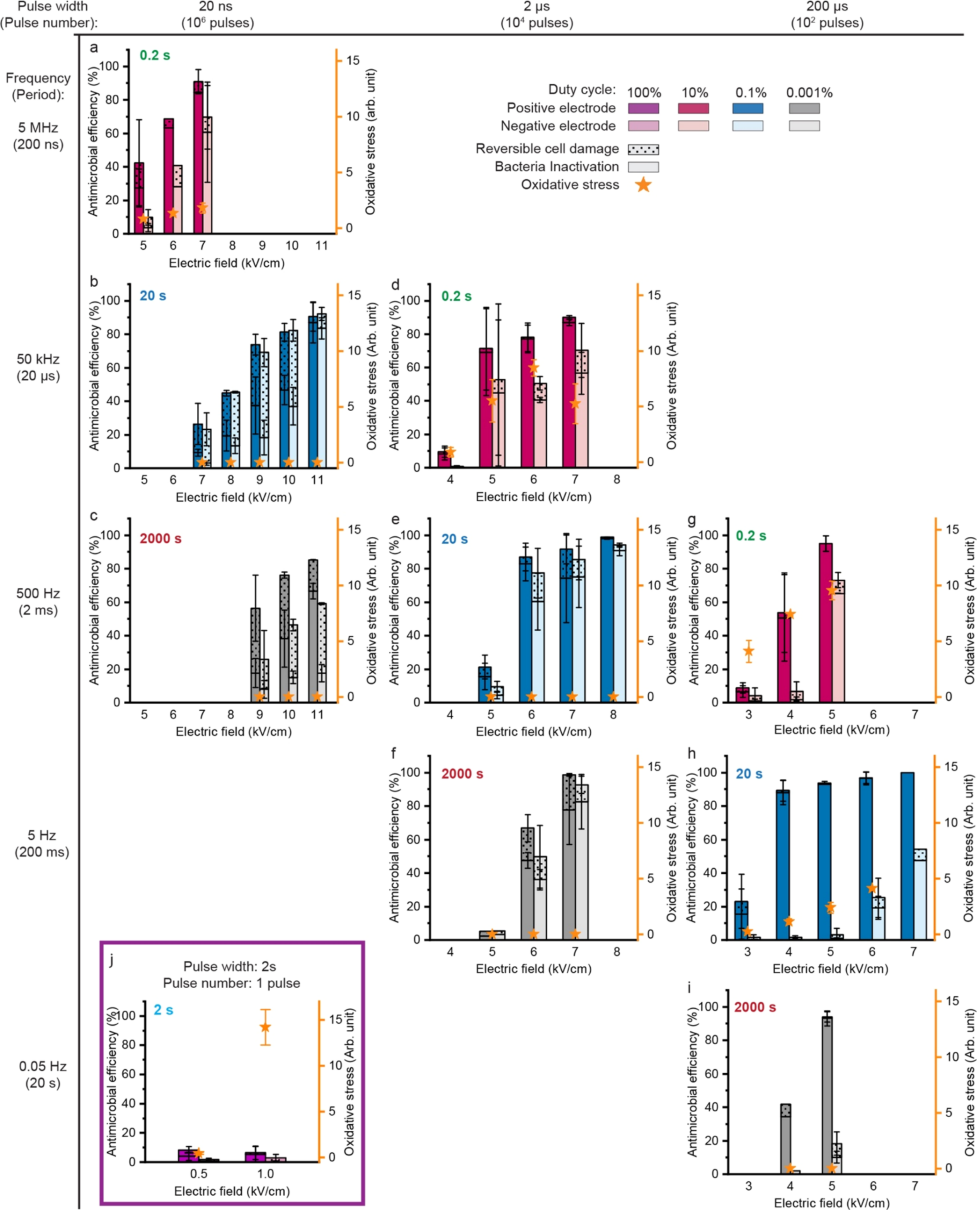
Performance of LEEFT under different electrical treatment conditions. The figures are arranged according to the pulse width (horizontal) and frequency/period (vertical). The results of DC treatment are shown in panel (j) separately. The effective treatment time is kept at 20 ms by adjusting the applied pulse number for all treatment conditions other than DC treatment. For DC treatment in panel (j), a single 2 s pulse is applied, making the effective treatment time 2 s. The total treatment time is calculated by the product of the period and pulse number, which is labeled at the upper left corner of each figure.

The LEEFT mechanism is tuned in DI water (0.5 μS/cm) between electroporation and oxidation by adjusting treatment conditions, i.e., the electric field strength, pulse width, and frequency (Figure 2). Reversible cell damage and permanent cell inactivation together represent the antimicrobial efficiency, which is determined by the double staining method using SYTOX Green and the PI stain and characterized by processing the microscopy images. The oxidative stress is characterized by processing DCFH-DA fluorescence (like the images shown in Figure 1). In each figure of Figure 2, a positive oxidative stress level indicates that electrochemical oxidation is induced. A low antimicrobial efficiency suggests that no effective electroporation occurs. This is because the antimicrobial efficiency is characterized by the percentage of nanowedges that can induce cell damage/inactivation at tips (defined in Section 2.3), which is a specific phenomenon of electroporation, as shown in Figure 1c but not in Figure 1e. Based on these criteria, the conditions can be categorized as electroporation-dominant (Figure 2b,c,e,f,i), oxidation-dominant (Figure 2j), and the combination of both (Figure 2a,d,g,h).

The trend of oxidation occurrence can be summarized from the figures. Briefly, oxidation increases along with the duty cycle (pulse width to period) and pulse width. At a relatively high duty cycle (10%), all three pulse widths induce oxidative stress (Figure 2a,d,g), even under a low electric field of 5 kV/cm with the ultrashort 20 ns pulses (Figure 2a). When DC is applied (i.e., 100% duty cycle), 2 s can induce significant oxidation at an electric field as low as 1 kV/cm (Figure 2j). A higher duty cycle is a higher pulse width to period ratio, which means a shorter interval between pulses, so the voltage built upon the electrical double layer may not be discharged completely. Since the pulses are delivered in a single direction, the voltage could accumulate after each pulse and reach the threshold to induce electrochemical reactions. Similarly, the intermediate species formed during a pulse could not diffuse away or be consumed in this short interval but instead accumulate, thus leading to the formation of reactive oxygen species.

It is intuitive that a longer pulse width contributes to oxidation since each pulse could be long enough to charge the electrical double layer to reach the oxidation threshold, as discussed in the introduction. At 0.1% duty cycle, oxidative stress is induced only with the longest pulse width, 200 μs (Figure 2h). No oxidative stress is detected at a 0.001% duty cycle under 200 μs pulses up to 5 kV/cm (Figure 2i), but it is still possible if the electric field keeps increasing.

In the next sections, we will discuss the mechanism as electroporation-dominant, oxidation-dominant, and the combination of both, respectively.

### 3.3 Electroporation as the Predominant Mechanism

The treatment conditions shown in Figure 2b,c,e,f,i are electroporation-dominant. It can be seen from all of these figures that the antimicrobial efficiency is strictly correlated to the electric field. A higher antimicrobial efficiency can be achieved by a stronger electric field when other parameters are kept the same. When the electric field is lower than 4 kV/cm, the antimicrobial efficiency is low under all conditions tested. To further confirm this, even longer pulses are tested. When 10 ms pulses are applied at 0.1% duty cycle (i.e., 10 s period), about 16% antimicrobial efficiency is achieved at 4 kV/cm, but bubbling (water electrolysis) is already induced (data not shown). These results indicate that electroporation has an electric field threshold, lower than which electroporation cannot be induced, no matter how long the pulse or treatment time is. At the same time, with a longer pulse width and a higher duty cycle, electrochemical oxidation usually dominates the process even with a low applied electric field.

Longer pulse widths contribute more to the antimicrobial efficiency than a higher frequency. Under the same frequency, a longer pulse width significantly increases the antimicrobial efficiency (Figure 2c vs e). However, under the same pulse width, a higher frequency does not increase the antimicrobial efficiency so significantly (Figure 2b vs c, or e vs f). This can be further confirmed when the same duty cycle is kept. With a duty cycle of 0.1 or 0.001%, a longer pulse width achieves a higher antimicrobial efficiency (Figure 2e vs b, or i vs f vs c), even though the frequency is reduced by the same ratio. For example, with a duty cycle of 0.1%, about 80% of the total antimicrobial efficiency (bacteria inactivation + reversible cell damage) is achieved at both electrodes under 2 μs pulses at 7 kV/cm (Figure 2e), while only 20% is achieved under 20 ns (Figure 2b).

The effects of the pulse width on the antimicrobial efficiency can be explained by the membrane charging mechanism. Charging the lipid bilayer membrane is like charging a capacitor in a circuit, which commonly requires a few micron seconds. Although in LEEFT, the cells at the nanowedge tip could be charged by the charges concentrated at the nanowedge tip very fast, longer pulses may induce more or bigger pores on the membrane, causing more severe cell damage and more inactivation. The frequency is not directly related to membrane charging. Each pulse charges the membrane independently, and the membrane can be fully discharged between pulses. Although increasing the frequency will not significantly improve the antimicrobial efficiency, it can reduce the total treatment time, thus improving the treatment efficiency.

### 3.4 Oxidation as the Predominant Mechanism

Electrochemical oxidation dominated the mechanism under DC for 2 s (Figure 2j). A very high oxidation level is detected with an electric field as low as 1 kV/cm. The strong oxidation is due to the high duty cycle (100%) and the long pulse width (2 s) if DC treatment is considered a single pulse. The antimicrobial efficiency at the nanowedge tip is low because no electroporation is induced at this low electric field. When oxidation is the predominant mechanism, the bacteria inactivation occurs randomly on the positive electrode surface (as shown in Figure 1e) instead of specifically at the nanowedge tips as that in electroporation (as shown in Figure 1c). Since the antimicrobial efficiency shown in the figure is characterized by the percentage of nanowedges that can kill bacteria at tips, the bacteria killed on the electrode surface are not counted. This can explain why the antimicrobial efficiency is low, although a decent number of cells are killed overall.

### 3.5 Combination of Electroporation and Oxidation

The antimicrobial efficiency can be enhanced by oxidation at the positive electrode; therefore, the efficiency of the positive electrode is generally higher than that of the negative electrode (Figure 2a,d,g,h). The antimicrobial efficiency also intuitively increases with the increase of the electric field and pulse width. What’s interesting is that when comparing Figure 2g,h, although the oxidation level is lower with a lower duty cycle (Figure 2h), the antimicrobial efficiency is not affected. This is probably because of the fact that at 0.1% duty cycle, the treatment has a longer total time, which is 20 s compared to 0.2 s for 10% duty cycle. Oxidation needs adequate time to make cell damage occur. Although the treatment finished within 0.2 s generates a significant amount of reactive species, they could diffuse away and be diluted after 0.2 s, while the longer treatment of 20 s can pose continuous damage on cells for a longer time.

It can be seen from the figures that the oxidative stress level is positively correlated to the electric field. Theoretically, it is determined more by the activation overpotential, but the gap between two electrodes can also have some effects. The oxidative stress detected by DCFH-DA is mainly caused by ROS, such as ·OH radical, generated during electrochemical reactions.^20,21^ Although ROS cannot be easily generated under DC on gold electrodes, it is possible that under short pulses with a high applied voltage, intermediate ROS are generated before oxygen evolution. The ROS generation is correlated to the current density, which is determined by the activation overpotential according to the Butler–Volmer equation. In addition, the resistance of the solution between two electrodes should also be significant, especially for a larger electrode gap and a lower solution conductivity since it obeys Ohm’s law. Therefore, when the same voltage is applied across electrodes with a larger electrode gap, the oxidation level is lower (Figure S5). In summary, a higher applied voltage (i.e., a higher applied electric field when the electrode distance is the same), smaller electrode distance, and higher conductivity can result in a higher current density and stronger oxidation.^22^ In addition to the electric field, the oxidation level is enhanced under a higher duty cycle (Figure 2g vs h) and with a longer pulse width (Figure 2a vs d vs g).

### 3.6 Overall Mechanism Tuning

**Figure 3:**
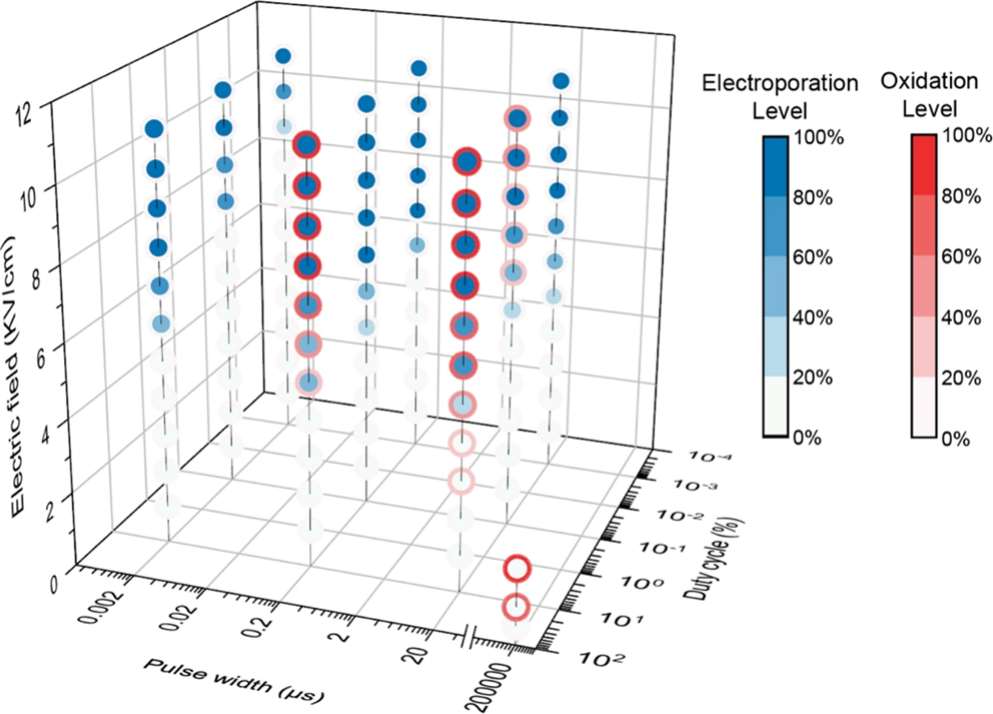
Distribution of electroporation and oxidation with the electric field strength, pulse width, and duty cycle in DI water. The blue dots and red circles represent the electroporation and oxidation levels, respectively. The electroporation levels are calculated based on linear equations achieved by fitting the total antimicrobial efficiency (reversible damage + bacteria inactivation) at the negative electrode with the electric field at each treatment condition, as shown in Figure 2. Only using the antimicrobial efficiency at the negative electrode is to rule out the effects of oxidation at the positive electrode. The oxidation levels are calculated based on linear equations achieved by fitting the measured oxidative stress with the electric field and then normalized by taking an oxidative stress of 20 as the highest level or 100%.

Electrochemical oxidation could occur simultaneously with electroporation in LEEFT, mainly controlled by the pulse width, duty cycle, and electric field strength (Figure 3). Collectively, a higher electric field, longer pulse width, and higher duty cycle represent stronger treatment conditions, which could achieve a higher antimicrobial efficiency and also lead to a higher level of oxidative stress. Electrochemical oxidation causes problems such as bubbling, generating byproducts, and electrode corrosion, thus deteriorating the quality of the water. Nevertheless, oxidation can also kill bacteria and help increase the antimicrobial efficiency (Figure 2a vs b). In addition, oxidation may help remove nucleic acids or other biomolecules released from cells as a result of electroporation, thus preventing potential horizontal gene transmission. Therefore, for applications that need to maintain a high liquid quality, such as drinking water treatment, the oxidation level should be controlled within an acceptable range to limit contamination. Under a duty cycle of 0.1% or less and a pulse width of 2 μs or less, oxidation could be largely inhibited, and electroporation dominates the mechanism for bacteria inactivation (Figure 3). There is still a big gap between the duty cycle of 0.1–10% and the pulse width of 2–200 μs, so we will do more comprehensive studies in the future with smaller gaps. Short pulses (e.g., 2 μs) applied at a moderate duty cycle (e.g., 0.1%) and a higher electric field (>6 kV/cm) can achieve a good antimicrobial efficiency by electroporation (Figures 2e and 3). For other applications where oxidation is acceptable, such as wastewater treatment, oxidative stress can be intentionally induced to enhance the bacteria inactivation efficiency. A relatively long pulse width (e.g., from 2 to 200 μs) and a higher duty cycle between 0.1 and 10% could be considered (Figure 3). Nevertheless, oxidation will dominate with further longer pulses (e.g., >200 μs) and a higher duty cycle (e.g., ≥10% and DC), which can induce strong oxidation even at a low electric field (e.g., ≤3 kV/cm) where electroporation does not occur yet.

### 3.7 Performance in the Medium with a Higher Conductivity

**Figure 4:**
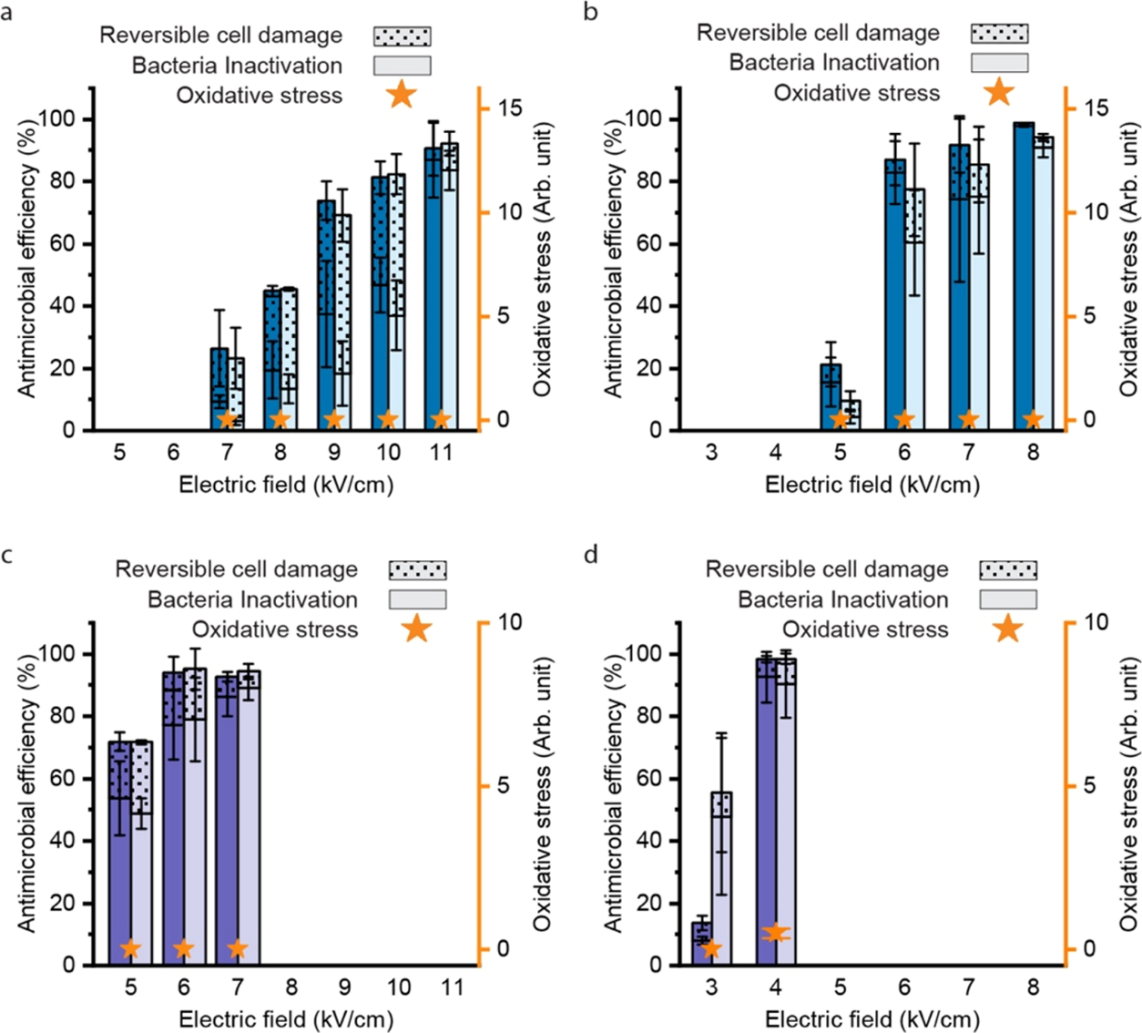
Performance of LEEFT in (a, b) DI water and (c, d) 6.7 mM Na_2_SO_4_ solution. (a, c) 20 ns pulses and (b, d) 2 μs pulses at 0.1% duty cycle. The conductivities of DI water and 6.7 mM Na_2_SO_4_ solution are 0.5 and 1500 μS/cm, respectively.

The conductivity of the extracellular medium could affect the efficiency of electroporation. The electric field threshold to kill bacteria decreases when the medium conductivity increases, so a lower electric field could be adequate to cause bacteria inactivation.^23^ Nevertheless, electrochemical reactions are more likely to be induced in solutions with a higher ionic strength. The antimicrobial efficiency in 6.7 mM Na_2_SO_4_ solution (1500 μS/cm) is compared with that in DI water (0.5 μS/cm) with 20 ns and 2 μs pulses, respectively (Figure 4). With 20 ns pulses, around 80% bacteria inactivation is achieved at 6 kV/cm in Na_2_SO_4_ solution, which equals that in DI water at 11 kV/cm. Under 2 μs pulses, 4 and 8 kV/cm achieve a similar antimicrobial efficiency in Na_2_SO_4_ solution and DI water, respectively. In Na_2_SO_4_ solution, no oxidation is detected up to 7 kV/cm with 20 ns pulses, but slight oxidation is detected at 4 kV/cm with 2 μs pulses. The conductivities of tap water, river water, and juice are around 50–800, 100–2000, and 2000–30 000 μS/cm, respectively. Therefore, compared with DI water, a better antimicrobial performance is expected to be achieved when drinking water or natural water is treated. Since there is a higher risk of generating oxidation with long pulses in media with a higher conductivity, using short pulses, such as nanosecond pulses, can be a good alternative.

### 3.8 Bacteria Inactivation Patterns under Different Mechanisms

**Figure 5:**
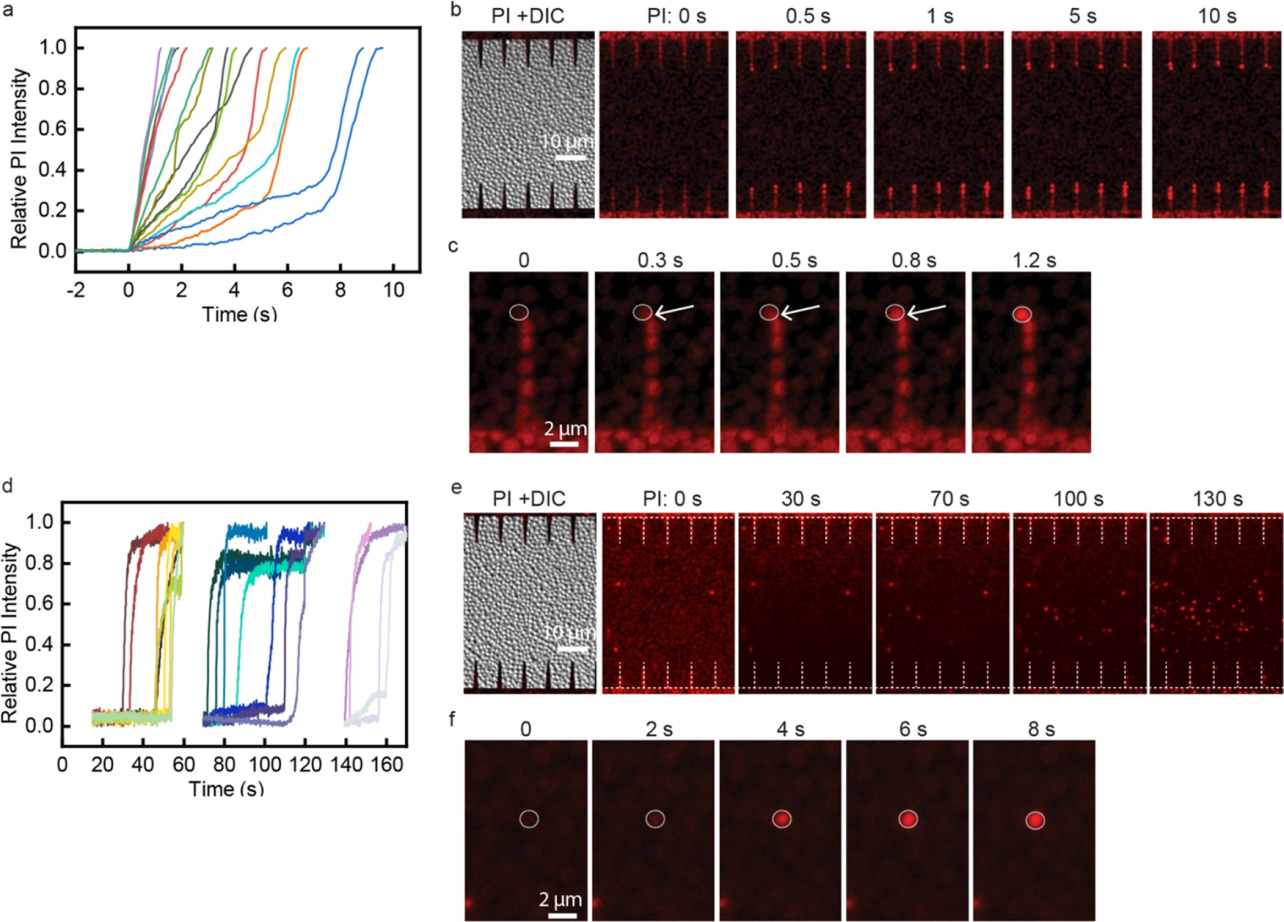
Bacteria inactivation pattern under electroporation and electrochemical oxidation. (a–c) Bacteria inactivation pattern by electroporation (10 000 2 μs pulses delivered at 8 kV/cm and 0.1% duty cycle). (d–f) Bacteria inactivation pattern by electrochemical oxidation (DC for 5 s at 0.5 kV/cm). (a, d) Changing of the relative PI intensity in inactivated cells. Each line represents the relative PI fluorescence intensity of a bacterial cell. The electrical treatment starts at 0 s. (b, e) Microscopy images of bacteria after treatment starts. Panel (a) shows the fluorescence of cells in panel (b), and panel (d) shows the fluorescence of cells in panel (e). The cells having faint instead of bright red color in panel (e) at 0 s are all live cells. The faint color is from the original fluorescence of PI stain molecules when they accumulate outside the cell membrane before binding to DNA. The fading of the color at 30 s is due to oxidation of the PI stain molecules. (c, f) Microscopy images of a single inactivated cell at the nanowedge tip (c) for electroporation and in the bulk region between two electrodes (f) for oxidation showing different dye diffusion patterns. The arrows indicate the place where the cell is close to the nanowedge tip and has cell damage.

Bacteria inactivation shows different patterns under different inactivation mechanisms. If inactivated by electroporation, the cells very close to the nanowedge tips start to show a PI fluorescence increase right after the pulses start, indicating an immediate pore formation and thus quick cell damage or inactivation (Figure 5a–c). The cell inactivation is completed within about 10 s, and almost no more cells will be inactivated after that. The bacteria inactivation pattern is significantly different under electrochemical oxidation (Figure 5d–f). After the treatment starts, cells cannot be killed immediately (Figure 5d). The PI stain molecules accumulated outside the cell membrane are first oxidized near the positive electrode, making the original color of the dye fade out (30 s, as shown in Figure 5e), which can also indicate that oxidation is induced. After about 30 s, random cells near the positive electrode start to be damaged/inactivated and show a fluorescence increase (Figure 5d,e). Although the treatment itself is only 2 s, the cell inactivation can randomly take place during more than 160 s after treatment (Figure 5d), which is much longer than 10 s for electroporation (Figure 5a). Overall, oxidation can kill bacteria at a larger region, but only around the positive electrode area and shows a delay time (∼30 s). Electroporation kills bacteria in a more refined region (only at nanowedge tips) but is efficient for both positive and negative electrodes and kills bacteria much faster only within a few seconds.

In addition to the overall cell inactivation pattern, the cell damage also shows difference at the single-cell level. For electroporation, the fluorescence of the PI stain starts from one point at the edge of the cell where it is adjacent to the nanowedge tip and then diffuses to the whole cell (Figure 5c). This is because the cell membrane adjacent to the nanowedge tip experiences the highest electric field; thus, pores tend to generate at this point first. For oxidation, cells are randomly attacked by oxidation species in the medium, thus creating damage all over the cell randomly, and no specific PI-entering point can be seen (Figure 5f).

## 4 Environmental Implication

Most of the current commonly used disinfection methods are chemical-based and rely on oxidation reactions. Comparing with chemical methods, the main advantage of physical approaches is the capability to minimize byproducts. Electroporation, an electrophysical mechanism, has been claimed as the main mechanism of LEEFT in most of the previous studies; however, electrochemical reactions (e.g., electrochemical oxidation) can also be induced by electrical pulses. Although usually unwanted due to drawbacks like generating byproducts, oxidation can kill bacteria, remove released biomolecules such as DNA, and enhance the overall disinfection efficiency. The two mechanisms, electroporation (the electrophysical mechanism) and oxidation (the electrochemical mechanism), can overcombine with each other under different treatment parameters. Different mechanisms may suit different applications. In the applications where byproducts should be strictly limited, like drinking water or liquid food disinfection, electroporation is the preferred mechanism. In some other applications where the disinfection byproducts are less concerned, like wastewater treatment, oxidation could be enhanced intentionally to improve the disinfection efficiency. Therefore, tuning LEEFT between electrophysical (electroporation) and electrochemical (oxidation) mechanisms is desired for different applications.

In this study, we found that oxidation could be significantly inhibited with low duty cycles and short pulse widths in LEEFT. Electroporation could only be induced when the electric field is above a certain threshold level, and a good antimicrobial efficiency could be achieved by electroporation with a relatively higher applied electric field. Combining electroporation and oxidation can significantly enhance the antimicrobial efficiency. The medium with a high conductivity can easily induce electrochemical reactions, but even without oxidation effects, the antimicrobial efficiency could be enhanced compared to DI water.

The experimental setup in this study, including using a pure gold electrode, DI water, or Na_2_SO_4_ solution, is the simplest and most basic condition. The trend obtained from this study can be extrapolated to other applications when different electrode materials and different solutions are used. The parameters tested in this work (*e.g*., pulse width and duty cycle) still have a large gap in between (100 times), which may be too big to capture the mechanism transition point. Smaller gaps will be tested in future studies. Using lab-on-a-chip devices possesses several advantages, such as enabling real-time observation and single-cell-level investigation. Nevertheless, the microenvironment on the chip could be different from that in bulk water, including the diffusion limitation and different properties between attached and suspended bacteria. Therefore, testing free-moving bacteria in small volumes of bulk water under a microscope using the operando investigation method is another future study direction.

To better represent real water, a more complex water matrix should be considered. For example, as a ubiquitous species in real water, chloride may cause electrochlorination during electric field treatment, which can further enhance the disinfection efficiency, but it may also generate unwanted byproducts. The effects of these species in real water should be considered and studied. Another way to represent real water is to choose other model microbial strains. *S. epidermidis* was chosen in this work mainly due to its round and regular shape for easier image processing of the data acquisition and also to rule out the effects of the irregular cell shape on the cell transmembrane voltage uniformity around the cell. Nevertheless, a single type of bacteria cannot represent the true disinfecting performance in real water since there are different types of bacteria and even virus and protozoa in real water.^24^ Therefore, a potential future research direction is to investigate different types of microbes, including Gram-positive and Gram-negative bacteria, viruses, protozoa, and algae, and to discuss the effects of the cell shape, size, cell wall structure, growth status, and cell revival under different mechanisms.

Within electrified water treatment approaches, electrochemical oxidation is still the most intensively discussed method due to its high efficiency and good versatility. Electroporation as a physical mechanism has its inherent merits such as low byproduct generation and thus is promising and worth investigating. Therefore, this work systematically discussed how to realize the two mechanisms in LEEFT, by either toggling the mechanism between the two or combining them together. The future research directions to enhance the application of LEEFT include improving the disinfection efficiency and reducing the cost and energy consumption. By combining electroporation with electrochemical oxidation or other additive oxidants in LEEFT, the overall disinfection performance could be further improved, making it a better choice for more water treatment scenarios.

## Data Availability

Data in addition to the ones shown in the article and the Supporting Information are available from the authors upon reasonable request. The MATLAB scripts for the data analysis are available from the authors upon reasonable request.

## Supporting Information Available

Digital photo and microscopy image of the lab-on-a-chip (Figure S1); the relationship of the applied electric field and the enhanced electric field at the nanowedge tip (Figure S2); a graphical representation of pulse parameters and terms (Figure S3); a schematic of the double staining method with SYTOX Green and PI stain (Figure S4); and the oxidative level on chips with different electrode distances (Figure S5) (PDF)
