# Supplementary material for: Tuning the Locally Enhanced Electric Field Treatment (LEEFT) between Electrophysical and Electrochemical Mechanisms for Bacteria Inactivation
Source: Environ Sci Technol. 2024 Aug 6;58(33):14875–85. doi: 10.1021/acs.est.4c00503 (PMC11339917; doi:10.1021/acs.est.4c00503)
Supplement: Supplementary file 1 — es4c00503_si_001.pdf [file es4c00503_si_001.pdf]

Supplementary information for

Tuning the Locally Enhanced Electric Field Treatment (LEEFT) Between Electrophysical and Electrochemical Mechanisms for Bacteria Inactivation

Ting Wang[1], Xing Xie[1, 2*]

[1]School of Civil and Environmental Engineering, Georgia Institute of Technology, Atlanta, Georgia 30332, United States

[2]Institute for Electronics and Nanotechnology, Georgia Institute of Technology, Atlanta, Georgia, 30332, United States

*To whom the correspondence should be addressed. xing.xie@ce.gatech.edu

Summary: 7 pages, 5 figures.

Table of Contents

1. Supplementary figures

Figure S1. A digital photo and microscopy image of the lab-on-a-chip device.

Figure S2. Relationship of applied (background) electric and the enhanced electric field at nanowedge tip.

Figure S3. Graphical representation of pulse parameters and terms.

Figure S4. A schematic of the double staining method with SYTOX Green and PI stain.

Figure S5. The oxidative level on chips with different electrode distance.

1. Supplementary figures

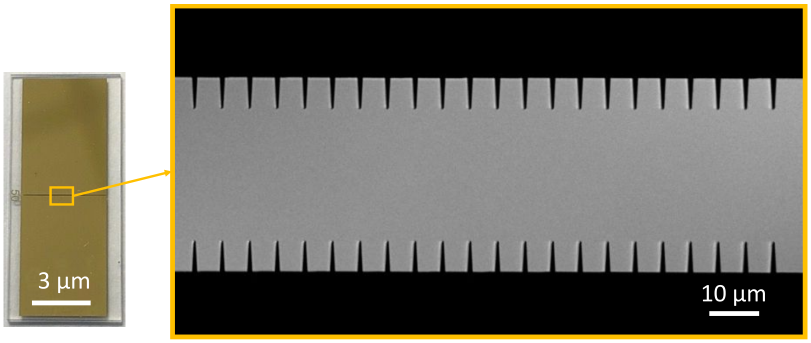

Figure S1. Digital photos and microscopy images of the lab-on-a-chip device with 50 µm gap between the two electrodes.

a b

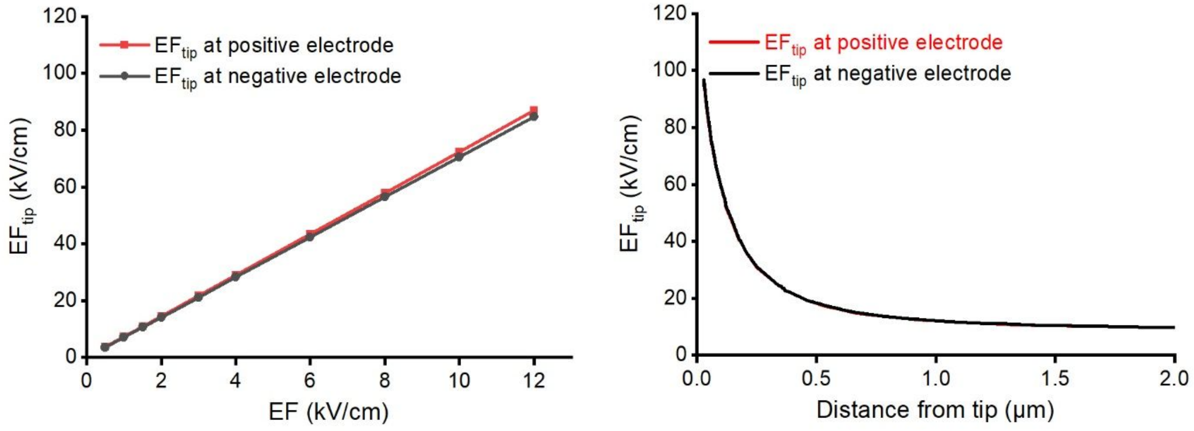

Figure S2. Relationship of applied (background) electric field (EF) and the enhanced electric field the nanowedge tip (EF_{tip}). (a) The EF_{tip} at 0.1 μm from the tip is enhanced about 7 times compared to the applied electric field. (b) EF_{tip} decreases with the increase of distance from tip (figure drawn at EF = 7 kV/cm).

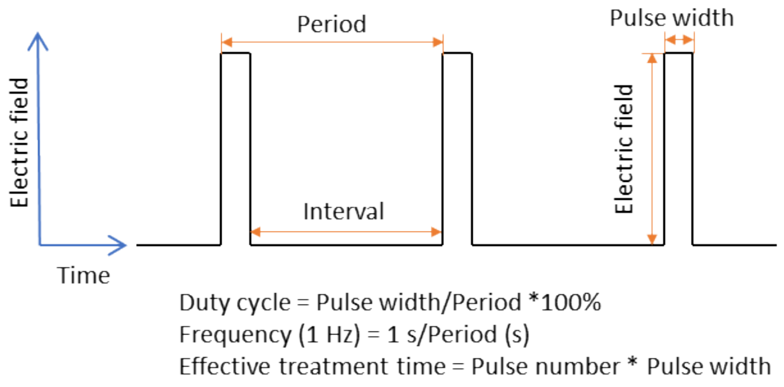

Figure S3. Graphical representation of pulse parameters and terms.

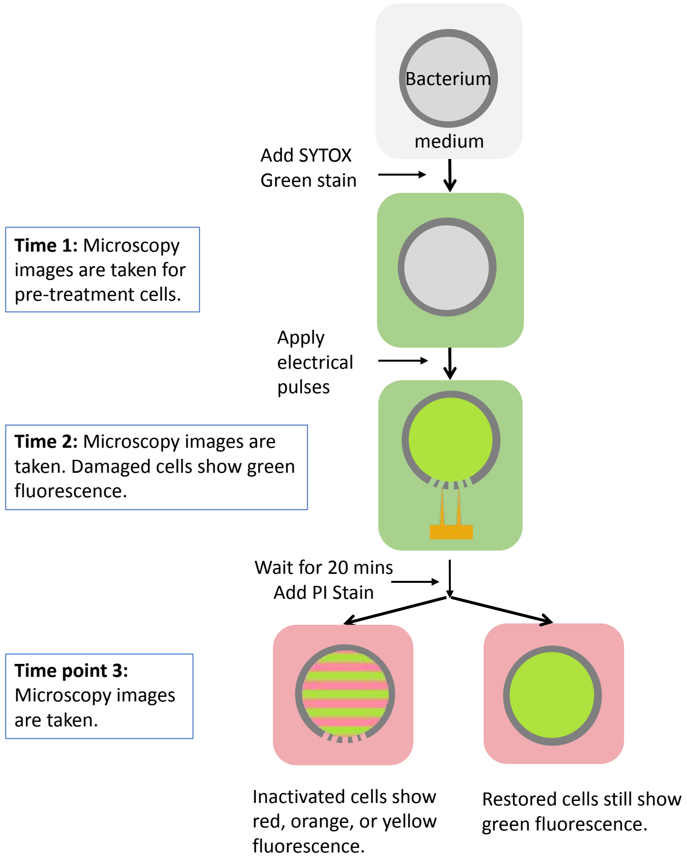

Figure S4. A schematic of the double staining method with SYTOX Green and PI stain. Before treatment, the bacteria immobilized on chip were immersed in a drop of DI water containing 5 µM live/dead cell viability stain SYTOX Green (Invitrogen). The cells damaged during the treatment show green fluorescence. Twenty minutes after the treatment, propidium iodide (PI, Invitrogen) was added at 15 µM to stain the inactivated cells, which would show a red, orange, or yellow fluorescence in the microscopy images. Reversible pores should already close within the 20 minutes window, so the cells stained with PI are considered to have permanent membrane damage and be inactivated.

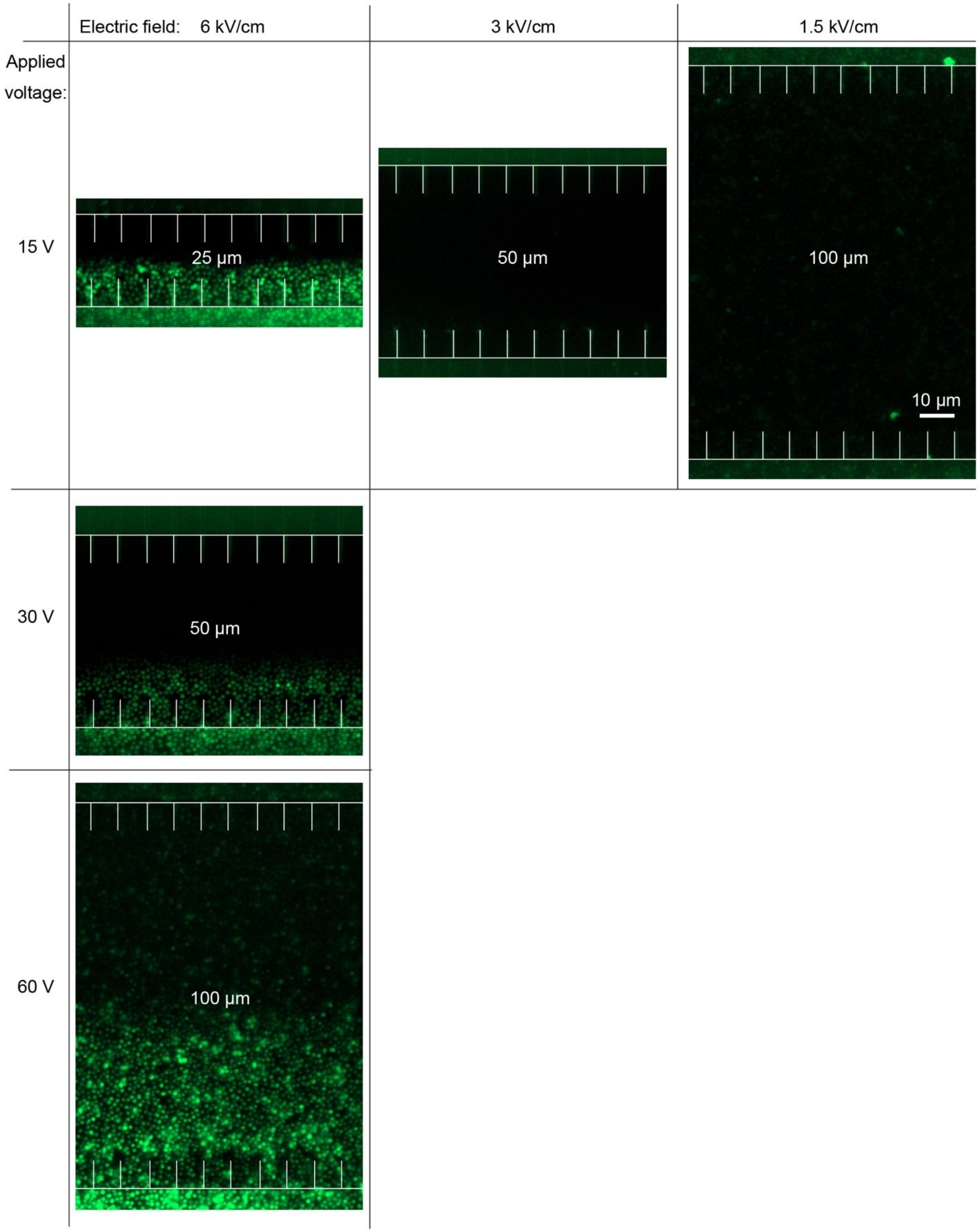

Figure S5. The oxidative level on chips with different electrode distance. The first row and column show results achieved under 15 V applied voltage or 6 kV/cm, respectively.
